# Quo Vadis Temporomandibular Disorders? By 2050, the Global Prevalence of TMD May Approach 44%

**DOI:** 10.3390/jcm14134414

**Published:** 2025-06-20

**Authors:** Grzegorz Zieliński

**Affiliations:** Department of Sports Medicine, Medical University of Lublin, 20-093 Lublin, Poland; grzegorz.zielinski@umlub.pl

**Keywords:** TMDs, temporomandibular disorders, meta-analysis, trends, prevalence, 2050, 2075, 2100, prediction, epidemiology

## Abstract

Background/Objectives: Currently, temporomandibular disorders (TMDs) represent a significant public health concern, affecting approximately 34% of the global population. The primary aim of this study was to determine the prevalence of TMDs in the year 2050. A secondary objective was to estimate the prevalence for the years 2030, 2075, and 2100. Methods: The methodology of a prognostic study was replicated and adapted to develop prevalence projections for TMDs, utilising the most recent meta-analysis of the global prevalence of temporomandibular disorders and analyses conducted within the R environment. Results: Projections indicate a gradual increase in the global prevalence of TMDs over the coming decades. In 2030, the estimated prevalence is 39% (95% confidence interval: 34–44%). This figure is expected to rise to 41% [36–46%] by 2040 and reach 44% [39–49%] by 2050. The upward trend continues, with projections suggesting a prevalence of 47% [42–52%] in 2075 and 49% [44–54%] by 2100. These data highlight a steadily increasing global burden of TMDs. Conclusions: By the year 2050, the global prevalence of TMDs is projected to reach 44%, which, according to estimates, corresponds to approximately 4,252,160,000 individuals. By 2030, 39% of the population is projected to experience TMDs. By 2075, the global TMD prevalence is expected to rise to 47%, and, by 2100, it could increase further to 49% of the global population. Urbanisation affects TMD prevalence in a region-dependent manner; a significant decrease was observed in Asia, while, in the Americas and Europe, the association was negligible. Globally, the lack of a clear impact of urbanisation on TMD occurrence suggests the influence of environmental and cultural factors.

## 1. Introduction

“Quo vadis, Domine?”—this question is not only a symbolic inquiry into spiritual direction, but also a profound reflection on humanity’s future in the face of suffering [1,2].

The Latin phrase “Quo vadis” means “Where are you going?” in English. In the context of medicine and biomedical sciences, the question “quo vadis?” refers to the search for ways to understand, diagnose, and treat diseases. It often arises in medical literature [3,4,5,6,7]. This question has also been posed in the present study in relation to temporomandibular disorders (TMDs).

TMDs refer to a group of dysfunctions involving the temporomandibular joint, the masticatory muscles, and adjacent structures, presenting with pain, limited jaw mobility, and joint sounds [8,9]. Currently, the global prevalence of TMDs is estimated at approximately 30% [10,11]. However, according to the latest meta-analysis (2024), there is geographical variation in the prevalence of TMDs: South America (47%), Asia (33%), Europe (29%), and North America (26%) [10].

The aetiology of TMDs is complex and involves multiple co-existing factors [12,13,14,15]. The development of TMDs may be influenced by biomechanical, neuromuscular, biopsychosocial, and biological elements [14]. Psychosocial factors, including stress, anxiety, and depression, also play a significant role, as they can exacerbate muscle tension and pain symptoms [16,17]. Recent studies have highlighted the comorbidity of bruxism and TMDs [18,19,20]. Bruxism, through the chronic overload of the masticatory muscles and temporomandibular joints, can lead to increased muscle tension and degenerative changes within the joint, significantly contributing to the development and exacerbation of TMD symptoms [18,19,20,21]. The global co-prevalence of bruxism and TMDs is around 17%, with regional variations similar to TMDs alone [10]: 70% in North America; 24% in South America; 14% in Europe; and 9% in Asia [18].

Due to its complex aetiology and high prevalence, TMDs represent a significant economic burden both for healthcare systems and for individual patients [22,23,24]. The treatment of TMDs involves a multidisciplinary approach, including conservative methods and, in more severe cases, invasive interventions [25,26,27,28]. Fundamental therapeutic strategies include occlusal splints, physiotherapy, and relaxation techniques aimed at reducing muscle tension. Pharmacotherapy is often used as an adjunct, including non-steroidal anti-inflammatory drugs, analgesics, and, sometimes, botulinum toxin to reduce masticatory muscle activity [25,27,29]. In chronic cases that are resistant to conservative treatment, surgical procedures may be considered [30,31].

Based on the data presented, it is evident that TMD constitutes a significant issue from epidemiological, diagnostic, and therapeutic perspectives, holding an important position within the interdisciplinary field of modern medicine. Therefore, this study was undertaken to analyse future trends in the prevalence of TMD. At the outset, it is important to emphasise that determining disease trends in medical research always carries a risk of error [32,33,34,35]. Nevertheless, even if forecasts are not entirely precise, they remain of great importance as they enable preventive actions and the more effective planning of resources such as hospital bed availability, medical staff, and medicine supplies. Accurate prognostic forecasts enhance the value of diagnosis, allowing for more efficient crisis management [36].

Moreover, identifying health trends allows for the development of prediction-based health policies, including increased preventive efforts such as educational campaigns, vaccination programmes, or the adaptation of medical infrastructure. A lack of such information may, in the long term, pose a greater threat to prevention than the potential inaccuracy of forecasts, which can be corrected over time. In the context of the COVID-19 pandemic, forecasts based on mathematical models played a crucial role in addressing the global health crisis [37,38,39].

Forecasting the incidence of TMDs may constitute a significant contribution to long-term planning at both the clinical and systemic levels. As predicted, an increase in the prevalence of TMDs could lead to a greater burden on healthcare systems, necessitating appropriate adjustments in training strategies, resource allocation, and the development of policies focused on prevention and early diagnosis [22,40,41,42,43].

Adopting a long-term perspective on this issue enables the identification of potential gaps in the availability and quality of care, while also promoting the development of integrated treatment models based on epidemiological analyses [44,45,46]. In this context, the question “Quo vadis?” in relation to TMDs takes on particular significance, acting as a stimulus for policymakers and clinical practitioners to initiate measures that prepare healthcare systems for future challenges.

To the best of the author’s knowledge, no studies have yet been published that forecast the future global prevalence of TMDs in a long-term framework. The projection presented in this work for the years 2030, 2050, 2075, and 2100 is, therefore, novel in nature and represents a meaningful expansion of the current body of knowledge. It may serve as a tool for enhancing the understanding of future epidemiological trends and predicting the potential burden on healthcare systems.

Incorporating long-term forecasts allows for the more precise planning of medical resources and the formulation of effective preventive and therapeutic strategies, which is of crucial importance both from a public health policy perspective and for clinical management within the field of TMD [47].

Therefore, in reference to the question posed in the title—Quo Vadis Temporomandibular Disorders?—a study was undertaken to determine the prevalence of TMDs in the year 2050. A secondary aim was to estimate the prevalence in the years 2030, 2075, and 2100.

## 2. Materials and Methods

The study design was registered with the Open Science Framework (OSF) under the identification number https://doi.org/10.17605/OSF.IO/U846T.

Based on the model proposed by Holden et al. [34], an attempt was made to estimate the hypothetical prevalence of TMDs in the years 2050 and 2100. The analysis covered six continents: North America, South America, Europe, Africa, Asia, and Australia. Data were grouped into three age categories, consistent with the classification used in the meta-analysis on TMD [10]: under 18 years, 18–60 years, and over 60 years.

Baseline prevalence data for TMDs in each age group were obtained from [10]—Supplementary Material S1. In cases where complete data were unavailable (partly for North America and entirely for Africa and Australia), global prevalence estimates by age group were used: 0–18 years (27%), 18–60 years (41%), and 60+ years (36%) [10]. These values were assumed to refer to the year 2020, in line with the cut-off date for the literature search in the meta-analysis [10], rounded to the nearest whole year (consistent with Holden et al. [34]).

### 2.1. Modelling and Estimation of Changes

The model developed by Holden et al. [34] was adapted for this analysis, employing linear regression to estimate future changes in TMD prevalence. This approach was deemed appropriate given the parallels between the increasing epidemiological trends observed in both myopia [34] and TMDs [10,11,48], as well as the shared multifactorial aetiology of these conditions (myopia [49,50,51] and TMDs [52,53,54]). The model incorporated current prevalence rates, changes in urbanisation (R^2^ = 0.07) [34], and the Human Development Index (HDI, R^2^ = 0.74) [55].

To translate the strength of the relationship between exposure variables and outcome, the hazard ratio (HR) was converted into a correlation coefficient (r), following a logistic regression and Cox-model-based approach [56,57]. Based on Bair et al. [58], a mean pseudo-R value of 0.57 was obtained. A 95% confidence interval was calculated for each forecast outcome [59,60].

### 2.2. Impact of Urbanisation on TMD Prevalence

Due to a lack of clear studies examining the effect of urbanisation on TMD prevalence, a systematic search of the PubMed and Scopus databases was conducted [21,61,62]. The keywords used were “TMD”, “temporomandibular disorder”, and “rural”, with no date restrictions. Of 190 identified articles, 66 abstracts were reviewed, and 18 full texts were analysed. Due to the inability to export data based on urban or rural regions, the following studies were excluded after full-text assessment: [63,64,65,66,67]. Ultimately, 13 studies [68,69,70,71,72,73,74,75,76,77,78,79,80] comparing TMD prevalence in urban and rural populations were included. The summary of the PICO standards (population, intervention, comparison, and outcome) [81,82] is found in Table 1.

Urbanisation rates (%) corresponding to the year of data collection were assigned based on literature values [55]. Studies were categorised by continent: America, Europe, and Asia. For each, linear regression (OLS—ordinary least squares) [83,84] was used to model the relationship between urbanisation (%) and TMD prevalence (%). Influential observations were removed using Cook’s distance (>4/n) [85,86]. Coefficients of determination (R^2^) were calculated, and regression coefficients were interpreted as the change in TMD prevalence per 1% increase in urbanisation.

These results were compared with the United Nations projections, indicating that 55% of the global population currently lives in cities, a figure expected to rise to 68% by 2050 [87]. As no forecast for the year 2100 was available, a linear extrapolation to 75% was adopted as a moderate and realistic assumption.

Assumed regression coefficients were as follows (Supplementary Material S2):

- For the Americas: −0.023 (i.e., a 0.023 percentage point decrease in TMD prevalence per 1% urbanisation increase);
- For Europe: ~0.000 (negligible effect of urbanisation);
- For Asia: −0.316 (i.e., a 0.316 percentage point decrease per 1% increase in urbanisation);
- Global coefficient: −0.0034. This indicates a negligible and likely clinically insignificant decrease of 0.0034 percentage points in TMD prevalence per 1% increase in urbanisation; yet, this was used in the analysis.

### 2.3. Annual Growth Model

To model a realistic rate of TMD prevalence increase, an exponential fit function was developed, based on the approach by Holden et al. [34]. The model accounts for a decreasing growth rate as prevalence approaches upper biological limits near 100%. The function was calibrated so that, for a baseline prevalence of 12% [58,88], it yielded an annual growth rate of exactly 3.5% [58] (Supplementary Material S3).

Final formula is as follows:

$${∆p}_{annual}=6.99*e^{-0.057*p_{2020}}$$

where:

-
${∆p}_{annual}$ = annual change in TMD prevalence (percentage points);
- *p*_2020_ = baseline TMD prevalence in the year 2020;
- *e* = Euler’s number (approx. 2.718) [89].

### 2.4. Age Adjustment Coefficients

To reflect age-related differences in growth rates, correction factors were developed. According to Bair et al. [58], annual prevalence growth was 2.9% (ages 18–24, n = 1421), 3.8% (ages 25–34, n = 736), and 4.7% (ages 35–44, n = 580). A weighted average annual growth rate of 3.52% for ages 18–44 was calculated and adopted as the reference for the broader 18–60 age group, in line with the meta-analysis range [10].

For the 0–18 group, a growth rate of 1.75% (correction factor: 0.50) was assumed, while, for the 60+ group, a rate of 1.3% (factor: 0.37) was applied. These values are based on empirical data and biological–demographic reasoning [10,11,21,34].

### 2.5. Model Limitations and Saturation Threshold

The adoption of a saturation threshold in forecasts is supported by both empirical evidence and methodological principles, in accordance with the standards of prognostic modelling and trend analysis. From a statistical perspective—particularly in linear regression applied to forecasting temporal values—the absence of anchoring the model within a realistic limit may result in the overestimation of future values and an illogical escalation of the trend [90,91,92,93].

Consequently, predictive analyses frequently employ the so-called saturation threshold [92,93,94], which defines the maximum level of a variable beyond which its growth rate diminishes, and forecasted values either stabilise or begin to decline.

Models of this nature have been known since the nineteenth century; for instance, the Belgian mathematician Pierre François Verhulst (1804–1849) developed the logistic model to describe constrained population growth in the context of finite environmental resources [90,91].

In the context of the epidemiology of TMDs, a 60% threshold has been grounded in empirical data: it represents twice the prevalence estimated by meta-analyses (~30% in 2020) [10,11], while simultaneously being corroborated by selected studies reporting values near or exceeding 60% in large population samples (n > 500) [95,96,97]. Although such values lie at the upper boundary of known data, they are observable in specific populations or under broad definitions of symptoms.

Therefore, the adoption of a 60% saturation threshold is methodologically justified and appropriate within the framework of modelling limited growth functions. This approach helps to avoid distortions arising from linear extrapolation and enhances the accuracy of predictions concerning the epidemiological dynamics of the phenomenon.

### 2.6. Omission of Gender Factor

Gender differences were not included in the model. A 2024 meta-analysis [10] found no statistically significant differences in TMD prevalence between men and women. Although female sex hormones may influence pain symptom severity, their role in TMD aetiology remains unclear [61,62].

### 2.7. Comparison with INED Data

The obtained results were compared with the exported data from the Institut national d’études démographiques (INED). The data referred to the projections available in May 2025, based on the Medium Scenario for the entire world [98].

### 2.8. Data Analysis

Analyses were conducted using the R Statistical language (version 4.1.1; R Core Team, 2021) on Windows 10 Pro 64 bit (build 19045), using the packages *metafor* (version 3.8.1; [99]), *dplyr* (version 1.1.2; [100]), *ggplot2* (version 3.4.0; [101]), readxl (version 1.4.5; [102]), scales (version 1.4.0; [103]), tidyr (version 1.3.1; [104]), and purr (version 1.0.4; [105]).

## 3. Results

**Figure 1:**
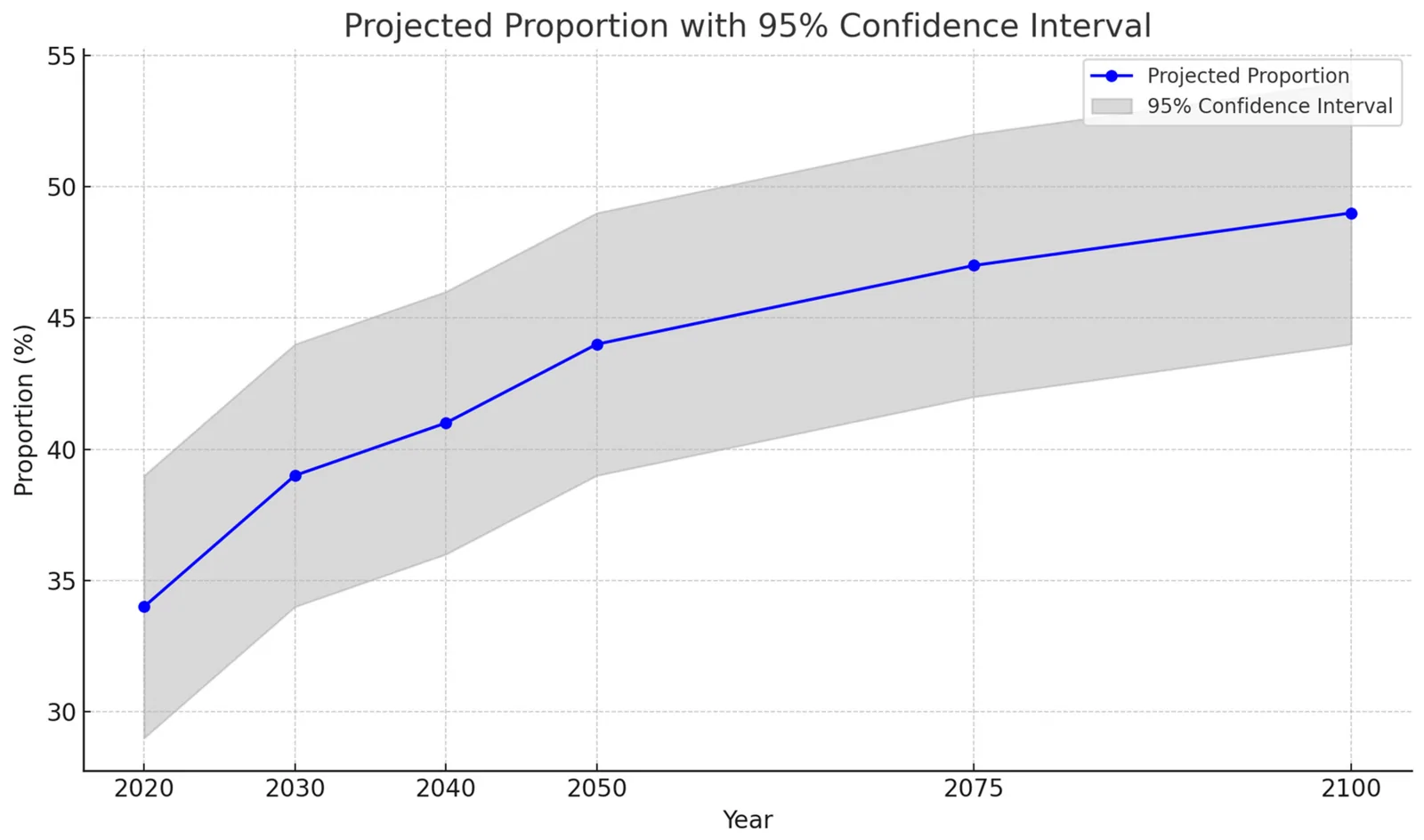
Graphical representation of the projected prevalence of TMD for the years 2050, 2075, and 2100, including 95% confidence intervals.

Based on the conducted analyses, it was observed that, by 2030, the global prevalence of TMDs will reach 39%. In 2040, it will exceed 40%, reaching 44% by 2050. In subsequent years, it is projected to be 47% in 2075 and 49% in 2100. Detailed data are presented in Table 2 and Figure 1.

The results were compared with INED data [98]. According to estimates, the global population is projected to reach 9664 million people by 2050, 10,250 million by 2075, and 10,180 million by 2100 [98]. A comparison of the percentage data (Table 2) with INED projections suggests that, by 2050, at least one symptom of TMD will be present in approximately 4252.16 million people worldwide. By 2075, this number is expected to rise to 4817.50 million, and, by 2100, to 4988.20 million people globally.

**Figure 2:**
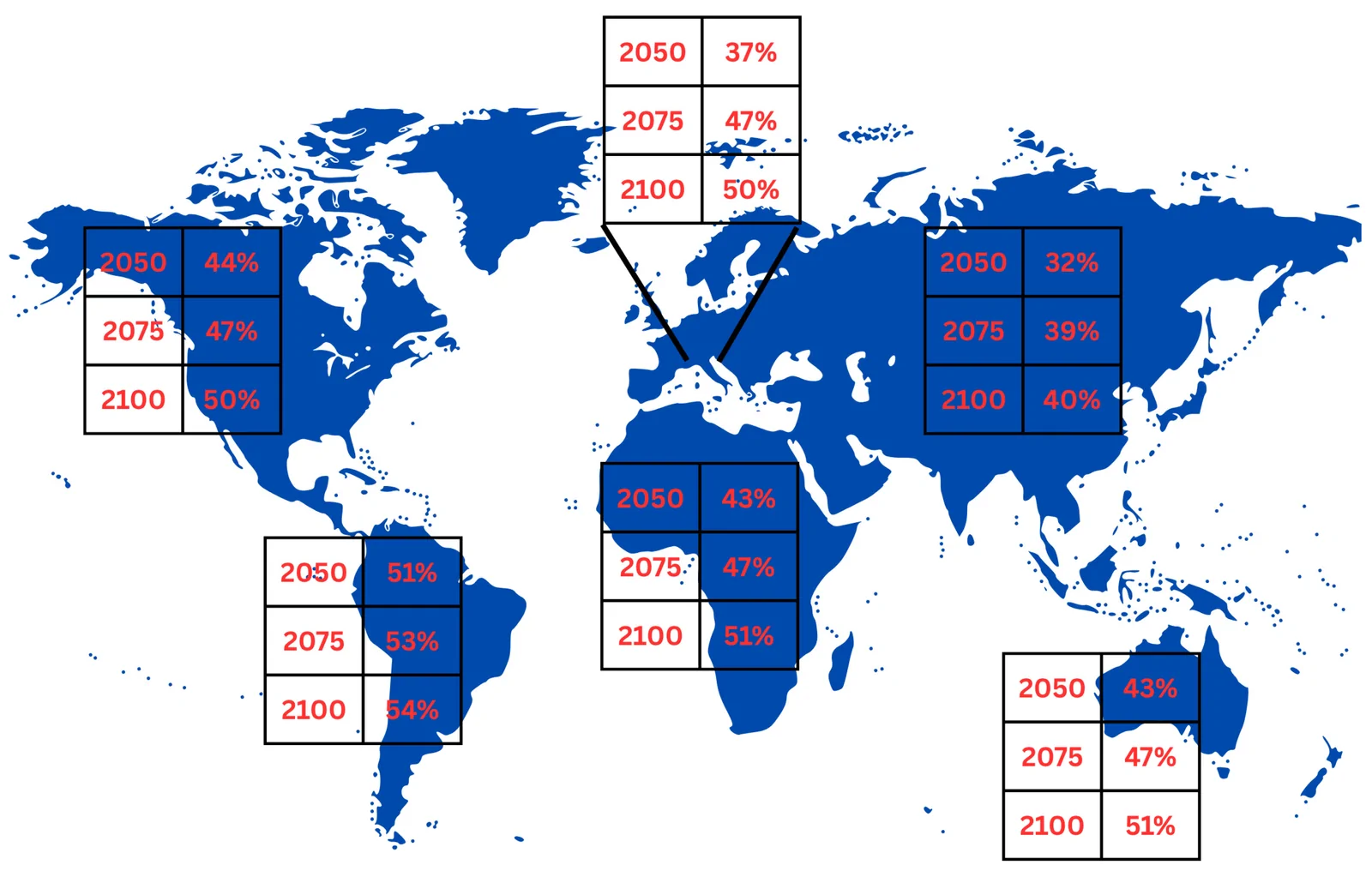
Graphical representation of TMD prevalence for the years 2050, 2075, and 2100 by continent.

In 2050, the estimated prevalence of TMDs in North America is projected to be 44%, while, in South America, it will reach 51%, representing the highest prevalence globally. In comparison, the prevalence in Europe is estimated at 37%, in Asia at 32%, in Africa at 43%, and in Australia at 43% (Figure 2). Due to a lack of detailed data for Africa and Australia, these figures have been averaged based on global estimates, as shown in Supplementary Material S1.

In the following decades, a systematic increase in TMD prevalence is expected. By 2100, South America, Africa, and Australia are projected to exceed a 50% prevalence of TMDs in the population. Europe is expected to approach 50%, and Asia will near 40% (Figure 2). Detailed data on age distribution are presented in Table 3.

## 4. Discussion

The primary aim of this study was to determine the prevalence of TMDs in the year 2050. A secondary objective was to estimate the prevalence for the years 2030, 2075, and 2100. In response to the titular question, “Quo Vadis Temporomandibular Disorders?”, the answer proposed is that TMDs are on a trajectory to exceed the 40% prevalence threshold worldwide. It is suggested that, by 2050, approximately 44% of the global population will be affected. This is expected to increase steadily, reaching 47% by 2075 and approaching 50% by the year 2100. The present article serves as a hypothesis which may be validated by future research.

TMD is an umbrella term that encompasses a range of diagnoses sharing common features such as pain, dysfunction, and restricted movement, although these conditions may have distinct aetiologies and require different therapeutic approaches [9,10,48,61,106]. It is important to emphasise that this analysis and its associated projections refer to TMDs in terms of this collective designation. The results presented here concern the occurrence of at least one TMD symptom within the studied population, based on data used as the foundation for the primary analysis [10].

Another key source instrumental in forming this analysis was the study by Holden et al. [34], which focused on myopia. It suggested that, by 2050, approximately 50% of the global population may experience myopia. Scientific literature indicates associations between refractive errors and TMDs, which provides a basis for comparing the findings of the two conditions [107,108,109,110,111,112]. Therefore, the 44% prevalence projected for 2050 in the current study may be considered a plausible and realistic hypothesis.

Even at present, some studies report TMD prevalence rates exceeding 40% [106,113,114], and, in certain populations, figures as high as 60% have been observed [96,115,116].

However, as noted in a recent meta-analysis concerning the global prevalence of TMDs [10], geographic variability and related factors play a crucial role. These include genetic predispositions [117,118,119], anthropological characteristics [14,120], disparities in access to healthcare [121,122], the incidence of bruxism [18,21], and population-specific differences in pain perception [123,124].

In light of these considerations, the assumption adopted in this study—that, by 2050, approximately 44% of the global population may exhibit at least one TMD symptom—appears to be both realistic and well-founded in the existing epidemiological data.

### 4.1. Geographic Differences in the Prevalence of TMD

The geographical variation in the prevalence of functional disorders of the masticatory system, known as TMDs, is a complex, multifactorial phenomenon. Among the key determinants of this variation are genetic predispositions, which are increasingly recognised as a significant component in the aetiopathogenesis of TMDs [117,118,119]. At the same time, environmental and cultural factors, as well as differing population exposures to risk factors such as bruxism, are also highlighted.

Recent meta-analyses point to a significant co-occurrence of bruxism and TMDs [18,19]. The global average prevalence of both conditions occurring together is approximately 17%, though this figure varies widely by region. In North America, the prevalence reaches as high as 70%; in South America, it is 24%; in Europe, 14%; and, in Asia, only 9% [18]. These differences may reflect both the distinct aetiological factors and variations in diagnostic methodologies employed in different regions [10].

One potential factor influencing these differences is the variation in pain perception and tolerance [123,124]. Global epidemiological studies covering 52 countries have shown that the average prevalence of chronic pain is 27.5%, with rates ranging from 9.9% to 50.3% depending on the country [125]. Furthermore, numerous scientific reports indicate differences in pain thresholds between ethnic groups. For instance, studies conducted in the United States have found that members of racial and ethnic minority groups tend to have a lower pain tolerance and greater sensitivity to pain stimuli [126].

Another important aspect is the accessibility and quality of healthcare, which can significantly affect epidemiological data. The literature highlights the substantial variation in healthcare spending between countries and regions, which does not always correlate with the quality of medical services or treatment outcomes [127]. Increasingly, research emphasises the need for systemic health policy interventions aimed at reducing regional disparities in access to healthcare services [128,129].

In summary, the observed geographical differences in the prevalence of TMDs may result from an interplay of biological, cultural, environmental, and systemic factors. This phenomenon requires further multi-centre epidemiological studies and an interdisciplinary research approach to deepen our understanding of the pathomechanisms of TMD and to develop more effective preventive and therapeutic strategies.

### 4.2. The 44% of the Population with TMD by 2050—What Comes Next?

Contemporary diagnostic and therapeutic approaches to the management of TMDs are demonstrating increasing efficacy and precision. Technological advancements, the expansion of medical knowledge, and the adoption of interdisciplinary strategies are contributing to a deeper understanding of the aetiopathogenesis of TMD and the optimisation of treatment pathways [27,130,131,132]. As outlined in the introduction to this paper, the current spectrum of available treatment modalities for TMDs encompasses both conservative strategies and more invasive interventions, selected individually based on the patient’s clinical phenotype [25,26,27,28].

Despite significant progress in this field, there remains a pressing need for continued intensive clinical and experimental research aimed at further refining diagnostic, therapeutic, and preventive methods. Particular emphasis should be placed on the development of strategies grounded in personalised medicine, taking into account genetic, environmental, and psychological factors that influence the clinical manifestation of TMD [8,10,16,17,117,119,130,132].

In the coming years, the advancement of artificial intelligence (AI) is likely to play a pivotal role, as its application in medicine becomes increasingly widespread [133,134,135]. Within the context of TMD, AI-based technologies may significantly enhance diagnostic processes, for example, through the analysis of imaging data (e.g., MRI, CBCT), symptom patterns, or data collected via digital tools [136,137]. Furthermore, machine-learning algorithms may, in the future, assist in therapeutic decision-making and the identification of high-risk patients, thereby enabling the implementation of early preventive interventions [133,134,135,136,137].

### 4.3. Limitations of the Forecast

Although this forecast is based on available data and reasonable assumptions, it is subject to a number of significant methodological limitations, which have been discussed in detail in the Introduction and the Materials and Methods sections. However, it is important to emphasise these issues explicitly here.

One of the main limitations of this analysis is the necessity of applying averaged global epidemiological values to estimate forecasts for entire continents such as Africa, Australia, and—in some cases—North America. This approach was necessitated by the lack of sufficiently detailed local source data, which represents a common challenge in modelling and prognostic studies of this kind [34].

An additional limitation involves the use of averaged urbanisation indices for the aforementioned regions, which may not fully capture the internal diversity within each continent. Despite these shortcomings, it should be noted that the substitution of missing data with estimated or averaged values is a widely accepted practice in population analyses and epidemiological forecasting, provided that such substitutions are properly documented and interpreted [34].

Another limitation of the analysis is the omission of sex-related differences. This decision was based on the findings of a recent meta-analysis, which did not reveal statistically significant differences in the prevalence of TMD between women and men [10]. Nevertheless, in light of the ongoing debate regarding the potential influence of hormonal factors on the pathophysiology of TMDs [61,62], as well as reports indicating a higher prevalence of the disorder among women, this omission should be acknowledged as a potential limitation that may influence future findings and their interpretation.

It should be clearly emphasised that the presented projection constitutes a prognostic hypothesis which—like any prediction based on statistical modelling—may or may not be confirmed by future empirical data. Its accuracy may be influenced by numerous dynamic and unpredictable external factors that were not accounted for in the present meta-analysis. Potential confounding variables include, among others, global health crises (e.g., pandemics), international conflicts, and political and regulatory changes (both those facilitating and those limiting access to healthcare services), as well as shifts in the demographic structure of the population. A significant impact may also arise from the advancements in the diagnosis and treatment of TMDs, including the development of new, more effective therapeutic approaches. In light of these considerations, the results presented herein should be interpreted as an approximation of a potential epidemiological trajectory rather than as a definitive or unchallengeable prediction.

In light of the above, the results presented here should be interpreted with caution—as an approximation of a potential trend, rather than a definitive prediction. They are intended to serve more as a starting point for further research than as a conclusive statement regarding the future prevalence of TMDs.

## 5. Conclusions

- By the year 2050, the global prevalence of TMDs is projected to reach 44%, which, according to estimates, corresponds to approximately 4,252,160,000 individuals.
- By 2030, 39% of the population is projected to experience TMDs. By 2075, the global TMD prevalence is expected to rise to 47%, and, by 2100, it could increase further to 49% of the global population.
- Urbanisation may exert varying effects on the prevalence of TMDs depending on the geographical region. The strongest association has been observed in Asian countries, where an increase in urbanisation levels correlated with a statistically significant decrease in TMD prevalence. In the Americas and Europe, this relationship was weak or not demonstrated. On a global scale, the analysis did not confirm a significant impact of urbanisation on TMD epidemiology, which may suggest the importance of environmental and cultural determinants in its aetiopathogenaesis.

## Figures and Tables

**Table 1:** PICO summary.

P (Population)	I (Intervention/Exposure)	C (Comparison)	O (Outcome)
Individuals from urban and rural populations studied for TMD prevalence.	Urbanisation (percentage of population living in urban areas).	Populations with different levels of urbanisation (rural vs urban regions).	Prevalence of temporomandibular disorders.

**Table 2:** Projected prevalence of TMDs for the years 2050, 2075, and 2100, including 95% confidence intervals.

Year	Projected Proportion	95% CI
2020	0.34 *	[0.29; 0.39] *
2030	0.39	[0.34; 0.44]
2040	0.41	[0.36; 0.46]
2050	0.44	[0.39; 0.49]
2075	0.47	[0.42; 0.52]
2100	0.49	[0.44; 0.54]

*—The data were added to illustrate the current state based on the work of [10].

**Table 3:** Projected TMD prevalence for the years 2050, 2075, and 2100 by age group and continent.

Region	Age Group	Projected Proportion	95% CI	Projected Proportion	95% CI	Projected Proportion	95% CI	Projected Proportion	95% CI
		2030		2050		2075		2100	
Africa	0–18	0.31	[0.26; 0.36]	0.40	[0.35; 0.45]	0.45	[0.40; 0.50]	0.49	[0.44; 0.54]
Africa	18–60	0.43	[0.38; 0.48]	0.49	[0.44; 0.54]	0.52	[0.47; 0.57]	0.55	[0.50; 0.60]
Africa	60+	0.37	[0.32; 0.42]	0.42	[0.37; 0.47]	0.45	[0.40; 0.50]	0.48	[0.43; 0.53]
Asia	0–18	0.29	[0.24; 0.34]	0.34	[0.29; 0.39]	0.37	[0.32; 0.42]	0.39	[0.34; 0.44]
Asia	18–60	0.42	[0.37; 0.47]	0.42	[0.37; 0.47]	0.44	[0.39; 0.49]	0.45	[0.40; 0.50]
Asia	60+	0.36	[0.31; 0.41]	0.35	[0.30; 0.40]	0.36	[0.31; 0.41]	0.37	[0.32; 0.42]
Australia	0–18	0.31	[0.26; 0.36]	0.40	[0.35; 0.45]	0.45	[0.40; 0.50]	0.49	[0.44; 0.54]
Australia	18–60	0.43	[0.38; 0.48]	0.49	[0.44; 0.54]	0.52	[0.47; 0.57]	0.55	[0.50; 0.60]
Australia	60+	0.37	[0.32; 0.42]	0.42	[0.37; 0.47]	0.45	[0.40; 0.50]	0.48	[0.43; 0.53]
Europe	0–18	0.25	[0.20; 0.30]	0.38	[0.33; 0.43]	0.44	[0.39; 0.49]	0.48	[0.43; 0.53]
Europe	18–60	0.43	[0.38; 0.48]	0.49	[0.44; 0.54]	0.52	[0.47; 0.57]	0.55	[0.50; 0.60]
Europe	60+	0.34	[0.29; 0.39]	0.40	[0.35; 0.45]	0.44	[0.39; 0.49]	0.47	[0.42; 0.52]
North America	0–18	0.38	[0.33; 0.43]	0.43	[0.38; 0.48]	0.46	[0.41; 0.51]	0.50	[0.45; 0.55]
North America	18–60	0.43	[0.38; 0.48]	0.48	[0.43; 0.53]	0.52	[0.47; 0.57]	0.54	[0.49; 0.59]
North America	60+	0.37	[0.32; 0.42]	0.41	[0.36; 0.46]	0.44	[0.39; 0.49]	0.47	[0.42; 0.52]
South America	0–18	0.35	[0.30; 0.40]	0.41	[0.36; 0.46]	0.45	[0.40; 0.50]	0.49	[0.44; 0.54]
South America	18–60	0.56	[0.51; 0.61]	0.56	[0.51; 0.61]	0.57	[0.52; 0.62]	0.57	[0.52; 0.62]
South America	60+	0.56	[0.51; 0.61]	0.56	[0.51; 0.61]	0.56	[0.51; 0.61]	0.56	[0.51; 0.61]

Detailed results of the analyses can be found in Supplementary Material S4.

## Data Availability

The original contributions presented in this study are included in the article/Supplementary Material. Further inquiries can be directed to the corresponding author.

## Supplementary Materials

The following supporting information can be downloaded at: https://www.mdpi.com/article/10.3390/jcm14134414/s1, Supplementary Material S1—Exported Data; Supplementary Material S2—Analysis of the Impact of Urbanisation on TMD Prevalence; Supplementary Material S3—Annual Growth Model; Supplementary Material S4—Detailed Results.

## References

[1] Szabolcsi R. (2020). Quo Vadis, Domine?. Technol. Soc. Sci. J..

[2] Song H.K. (2023). Quo Vadis Experimental Structural Biology?. Mol. Cells.

[3] Hazan Ben-Menachem R., Pines O., Saada A. (2024). Mitochondrial Derived Vesicles-Quo Vadis?. FEBS J..

[4] Gonzalez R.J., von Andrian U.H. (2022). Quo Vadis, Neutrophil?. Cell.

[5] Daubner-Bendes R., Kovács S., Niewada M., Huic M., Drummond M., Ciani O., Blankart C.R., Mandrik O., Torbica A., Yfantopoulos J. (2020). Quo Vadis HTA for Medical Devices in Central and Eastern Europe? Recommendations to Address Methodological Challenges. Front. Public Health.

[6] Zeidler H., Hudson A.P. (2022). Quo Vadis Reactive Arthritis?. Curr. Opin. Rheumatol..

[7] Miettinen O.S. (2004). Epidemiology: Quo Vadis?. Eur. J. Epidemiol..

[8] Zieliński G., Gawda P. (2025). Defining Effect Size Standards in Temporomandibular Joint and Masticatory Muscle Research. Med. Sci. Monit..

[9] Osiewicz M., Ciapała B., Bolt K., Kołodziej P., Więckiewicz M., Ohrbach R. (2024). Diagnostic Criteria for Temporomandibular Disorders (DC/TMD): Polish Assessment Instruments. Dent. Med. Probl..

[10] Zieliński G., Pająk-Zielińska B., Ginszt M. (2024). A Meta-Analysis of the Global Prevalence of Temporomandibular Disorders. J. Clin. Med..

[11] Valesan L.F., Da-Cas C.D., Réus J.C., Denardin A.C.S., Garanhani R.R., Bonotto D., Januzzi E., de Souza B.D.M. (2021). Prevalence of Temporomandibular Joint Disorders: A Systematic Review and Meta-Analysis. Clin. Oral Investig..

[12] Görürgöz C., İçen M., Kurt M.H., Aksoy S., Bakırarar B., Rozylo-Kalinowska I., Orhan K. (2023). Degenerative Changes of the Mandibular Condyle in Relation to the Temporomandibular Joint Space, Gender and Age: A Multicenter CBCT Study. Dent. Med. Probl..

[13] Seweryn P., Orzeszek S.M., Waliszewska-Prosół M., Jenča A., Osiewicz M., Paradowska-Stolarz A., Winocur-Arias O., Ziętek M., Bombała W., Więckiewicz M. (2023). Relationship between Pain Severity, Satisfaction with Life and the Quality of Sleep in Polish Adults with Temporomandibular Disorders. Dent. Med. Probl..

[14] Chisnoiu A.M., Picos A.M., Popa S., Chisnoiu P.D., Lascu L., Picos A., Chisnoiu R. (2015). Factors Involved in the Etiology of Temporomandibular Disorders—A Literature Review. Clujul Med..

[15] Osiewicz M., Ciapała B. (2024). Is Malocclusion a Predictor of Pain in Patients Suffering from TMD Pain?. Dent. Med. Probl..

[16] Fillingim R.B., Ohrbach R., Greenspan J.D., Knott C., Diatchenko L., Dubner R., Bair E., Baraian C., Mack N., Slade G.D. (2013). Psychological Factors Associated with Development of TMD: The OPPERA Prospective Cohort Study. J. Pain.

[17] Pihut M.E., Kostrzewa-Janicka J., Orczykowska M., Biegańska-Banaś J., Gibas-Stanek M., Gala A. (2024). Initial Assessment of the Psycho-Emotional State of Patients with Temporomandibular Disorders: A Pilot Study. Dent. Med. Probl..

[18] Zieliński G., Pająk-Zielińska B., Pająk A., Wójcicki M., Litko-Rola M., Ginszt M. (2025). Global Co-Occurrence of Bruxism and Temporomandibular Disorders: A Meta-Regression Analysis. Dent. Med. Probl..

[19] Mortazavi N., Tabatabaei A.H., Mohammadi M., Rajabi A. (2023). Is Bruxism Associated with Temporomandibular Joint Disorders? A Systematic Review and Meta-Analysis. Evid. Based Dent..

[20] Cigdem Karacay B., Sahbaz T. (2023). Investigation of the Relationship between Probable Sleep Bruxism, Awake Bruxism and Temporomandibular Disorders Using the Diagnostic Criteria for Temporomandibular Disorders (DC/TMD). Dent. Med. Probl..

[21] Zieliński G., Pająk A., Wójcicki M. (2024). Global Prevalence of Sleep Bruxism and Awake Bruxism in Pediatric and Adult Populations: A Systematic Review and Meta-Analysis. J. Clin. Med..

[22] Slade G., Durham J., Yost O., Liverman C.T., English R., Mackey S., Bond E.C., National Academies of Sciences, Engineering, and Medicine, Health and Medicine Division, Board on Health Care Services, Board on Health Sciences Policy, Committee on Temporomandibular Disorders (TMDs): From Research Discoveries to Clinical Treatment (2020). Prevalence, Impact, and Costs of Treatment for Temporomandibular Disorders. Temporomandibular Disorders: Priorities for Research and Care.

[23] White B.A., Williams L.A., Leben J.R. (2001). Health Care Utilization and Cost among Health Maintenance Organization Members with Temporomandibular Disorders. J. Orofac. Pain.

[24] Cho J.-H., Kim K.-W., Kim H., Shin W.-C., Kim M., Kim J., Kim M.-Y., Cho H.-W., Ha I.-H., Lee Y.J. (2025). Effectiveness and Cost-Effectiveness of Chuna Manual Therapy for Temporomandibular Disorder: A Randomized Clinical Trial. PLoS ONE.

[25] González-Sánchez B., García Monterey P., Ramírez-Durán M.d.V., Garrido-Ardila E.M., Rodríguez-Mansilla J., Jiménez-Palomares M. (2023). Temporomandibular Joint Dysfunctions: A Systematic Review of Treatment Approaches. J. Clin. Med..

[26] Tok Ö.N., Yurttaş A.N., Taş S. (2024). YouTube Videos as an Information Source about Exercises for Temporomandibular Disorders. Dent. Med. Probl..

[27] Gauer R.L., Semidey M.J. (2015). Diagnosis and Treatment of Temporomandibular Disorders. Am. Fam. Physician.

[28] Gębska M., Dalewski B., Pałka Ł., Kiczmer P., Kołodziej Ł. (2024). Kinesio Taping as an Alternative Therapy for Limited Mandibular Mobility with Pain in Female Patients with Temporomandibular Disorders: A Randomized Controlled Trial. Dent. Med. Probl..

[29] Delcanho R., Val M., Guarda Nardini L., Manfredini D. (2022). Botulinum Toxin for Treating Temporomandibular Disorders: What Is the Evidence?. J. Oral Facial Pain Headache.

[30] Dimitroulis G. (2018). Management of Temporomandibular Joint Disorders: A Surgeon’s Perspective. Aust. Dent. J..

[31] Sidebottom A.J. (2024). Current Thinking in Open Temporomandibular Joint Surgery. Is This Still Indicated in the Management of Articular Temporomandibular Joint Disorder?. Br. J. Oral Maxillofac. Surg..

[32] GBD 2021 US Obesity Forecasting Collaborators (2024). National-Level and State-Level Prevalence of Overweight and Obesity Among Children, Adolescents, and Adults in the USA, 1990-2021, and Forecasts up to 2050. Lancet.

[33] Hou Q., Li X., Ma H., Fu D., Liao A. (2025). A Systematic Epidemiological Trends Analysis Study in Global Burden of Multiple Myeloma and 29 Years Forecast. Sci. Rep..

[34] Holden B.A., Fricke T.R., Wilson D.A., Jong M., Naidoo K.S., Sankaridurg P., Wong T.Y., Naduvilath T.J., Resnikoff S. (2016). Global Prevalence of Myopia and High Myopia and Temporal Trends from 2000 through 2050. Ophthalmology.

[35] Fernandes G.V.D.O., Martins B.G.D.S., Fraile J.F. (2024). Revisiting Peri-Implant Diseases in Order to Rethink the Future of Compromised Dental Implants: Considerations, Perspectives, Treatment, and Prognosis. Dent. Med. Probl..

[36] Croft P., Dinant G.-J., Coventry P., Barraclough K. (2015). Looking to the Future: Should ‘Prognosis’ Be Heard as Often as ‘Diagnosis’ in Medical Education?. Educ. Prim. Care.

[37] Wynants L., Van Calster B., Collins G.S., Riley R.D., Heinze G., Schuit E., Bonten M.M.J., Damen J.A.A., Debray T.P.A., De Vos M. (2020). Prediction Models for Diagnosis and Prognosis of Covid-19: Systematic Review and Critical Appraisal. BMJ.

[38] Mateu L., Tebe C., Loste C., Santos J.R., Lladós G., López C., España-Cueto S., Toledo R., Font M., Chamorro A. (2023). Determinants of the Onset and Prognosis of the Post-COVID-19 Condition: A 2-Year Prospective Observational Cohort Study. Lancet Reg. Health Eur..

[39] Zhou B., Kojima S., Kawamoto A., Fukushima M. (2021). COVID-19 Pathogenesis, Prognostic Factors, and Treatment Strategy: Urgent Recommendations. J. Med. Virol..

[40] Yost O., Liverman C.T., English R., Mackey S., Bond E.C., National Academies of Sciences, Engineering, and Medicine, Health and Medicine Division, Board on Health Care Services, Board on Health Sciences Policy, Committee on Temporomandibular Disorders (TMDs): From Research Discoveries to Clinical Treatment (2020). Individual and Societal Burden of TMDs. Temporomandibular Disorders: Priorities for Research and Care.

[41] Seo H., Jung B., Yeo J., Kim K.-W., Cho J.-H., Lee Y.J., Ha I.-H. (2020). Healthcare Utilisation and Costs for Temporomandibular Disorders: A Descriptive, Cross-Sectional Study. BMJ Open.

[42] da Silva Z.A., Melo W.W.P., Ferreira H.H.N., Lima R.R., Souza-Rodrigues R.D. (2023). Global Trends and Future Research Directions for Temporomandibular Disorders and Stem Cells. J. Funct. Biomater..

[43] Ohrbach R., Dworkin S.F. (2016). The Evolution of TMD Diagnosis: Past, Present, Future. J. Dent. Res..

[44] Yost O., Liverman C.T., English R., Mackey S., Bond E.C., National Academies of Sciences, Engineering, and Medicine, Health and Medicine Division, Board on Health Care Services, Board on Health Sciences Policy, Committee on Temporomandibular Disorders (TMDs): From Research Discoveries to Clinical Treatment (2020). Improving TMD Health Care: Practice, Education, Access, and Coverage. Temporomandibular Disorders: Priorities for Research and Care.

[45] Deshmukh S.S., Kulkarni M.M., Bhatia A.P., Assiri K.I., Suleman G., Chaturvedi M., Alshahrani A.Y., Yaqoob A., Baig F.A.H., Khader M.A. (2025). Dental Practitioners Behaviour, Perspective and Treatment Approach towards Management of TMJ Disorders—An Observational Study. Technol. Health Care.

[46] Zhao R., Xiong X., Li Z., Zhang L., Yang H., Ye Z. (2025). Recent Advances and Educational Strategies in Diagnostic Imaging for Temporomandibular Disorders: A Narrative Literature Review. Front. Neurol..

[47] Soyiri I.N., Reidpath D.D. (2013). An Overview of Health Forecasting. Environ. Health Prev. Med..

[48] Schiffman E., Ohrbach R., Truelove E., Look J., Anderson G., Goulet J.-P., List T., Svensson P., Gonzalez Y., Lobbezoo F. (2014). Diagnostic Criteria for Temporomandibular Disorders (DC/TMD) for Clinical and Research Applications: Recommendations of the International RDC/TMD Consortium Network and Orofacial Pain Special Interest Group. J. Oral Facial Pain Headache.

[49] Kohanim S., Boelman B. (2022). The Multifactorial Etiology of Myopia. J. Stud. Res..

[50] Huang H.-M., Chang D.S.-T., Wu P.-C. (2015). The Association between Near Work Activities and Myopia in Children-A Systematic Review and Meta-Analysis. PLoS ONE.

[51] Cooper J., Tkatchenko A.V. (2018). A Review of Current Concepts of the Etiology and Treatment of Myopia. Eye Contact Lens.

[52] Kapos F.P., Exposto F.G., Oyarzo J.F., Durham J. (2020). Temporomandibular Disorders: A Review of Current Concepts in Aetiology, Diagnosis and Management. Oral Surg..

[53] Greene C.S. (2001). The Etiology of Temporomandibular Disorders: Implications for Treatment. J. Orofac. Pain.

[54] Sharma S., Gupta D.S., Pal U.S., Jurel S.K. (2011). Etiological Factors of Temporomandibular Joint Disorders. Natl. J. Maxillofac. Surg..

[55] United Nations Human Development Index.

[56] Chinn S. (2000). A Simple Method for Converting an Odds Ratio to Effect Size for Use in Meta-Analysis. Stat. Med..

[57] Goerdten J., Carrière I., Muniz-Terrera G. (2020). Comparison of Cox Proportional Hazards Regression and Generalized Cox Regression Models Applied in Dementia Risk Prediction. Alzheimers Dement. Transl. Res. Clin. Interv..

[58] Bair E., Brownstein N.C., Ohrbach R., Greenspan J.D., Dubner R., Fillingim R.B., Maixner W., Smith S., Diatchenko L., Gonzalez Y. (2013). Study Protocol, Sample Characteristics, and Loss to Follow-Up: The OPPERA Prospective Cohort Study. J. Pain Off. J. Am. Pain Soc..

[59] O’Brien S.F., Yi Q.L. (2016). How Do I Interpret a Confidence Interval?. Transfusion.

[60] Andrade C. (2023). How to Understand the 95% Confidence Interval Around the Relative Risk, Odds Ratio, and Hazard Ratio: As Simple as It Gets. J. Clin. Psychiatry.

[61] Zieliński G., Pająk-Zielińska B. (2024). Association between Estrogen Levels and Temporomandibular Disorders: An Updated Systematic Review. Int. J. Mol. Sci..

[62] Berger M., Szalewski L., Bakalczuk M., Bakalczuk G., Bakalczuk S., Szkutnik J. (2015). Association between Estrogen Levels and Temporomandibular Disorders: A Systematic Literature Review. Menopause Rev. Menopauzalny.

[63] Michalak M., Paulo M., Bożyk A., Zadrożny Ł., Wysokińska-Miszczuk J., Michalak I., Borowicz J. (2013). Incidence of Abnormalities in Temporomandibular Joints in a Population of 1,100 Urban and Rural Patients Lacking Teeth and Other Parafunctions in 2003-2008. An International Problem. Ann. Agric. Environ. Med. AAEM.

[64] Montero J., Llodra J.-C., Bravo M. (2018). Prevalence of the Signs and Symptoms of Temporomandibular Disorders Among Spanish Adults and Seniors According to Five National Surveys Performed Between 1993 and 2015. J. Oral Facial Pain Headache.

[65] Mejersjö C., Ovesson D., Mossberg B. (2016). Oral Parafunctions, Piercing and Signs and Symptoms of Temporomandibular Disorders in High School Students. Acta Odontol. Scand..

[66] Simangwa L.D., Johansson A.-K., Johansson A., Minja I.K., Åstrøm A.N. (2020). Oral Impacts on Daily Performances and Its Socio-Demographic and Clinical Distribution: A Cross-Sectional Study of Adolescents Living in Maasai Population Areas, Tanzania. Health Qual. Life Outcomes.

[67] Fabian F.M., and Mumghamba E.G.S. (2008). Risk Factors for Signs and Symptoms of TMD in a Rural Adult Southeast Tanzanian Population. CRANIO®.

[68] Restrepo C., Ortiz A.M., Henao A.C., Manrique R. (2021). Association between Psychological Factors and Temporomandibular Disorders in Adolescents of Rural and Urban Zones. BMC Oral Health.

[69] Costa M.J.F., Lins C.A.d.A., de Macedo L.P.V., de Sousa V.P.S., Duque J.A., de Souza M.C. (2019). Clinical and Self-Perceived Oral Health Assessment of Elderly Residents in Urban, Rural, and Institutionalized Communities. Clin. Sao Paulo Braz..

[70] Goddard G., Karibe H. (2002). TMD Prevalence in Rural and Urban Native American Populations. CRANIO®.

[71] Nilsson I.-M., List T., Drangsholt M. (2005). Prevalence of Temporomandibular Pain and Subsequent Dental Treatment in Swedish Adolescents. J. Orofac. Pain.

[72] Østensjø V., Moen K., Storesund T., Rosén A. (2017). Prevalence of Painful Temporomandibular Disorders and Correlation to Lifestyle Factors among Adolescents in Norway. Pain Res. Manag..

[73] Kim D., Ko S.-G., Lee E.-K., Jung B. (2019). The Relationship between Spinal Pain and Temporomandibular Joint Disorders in Korea: A Nationwide Propensity Score-Matched Study. BMC Musculoskelet. Disord..

[74] Shenoy R.P., Agrawal R., Abdul Salam T.A., Prashanth Shenoy K. (2020). Screening for Temporomandibular Disorders and Other Oral Conditions among Adolescents in Mangaluru Taluk. World J. Dent..

[75] Hongxing L., Astrøm A.N., List T., Nilsson I.-M., Johansson A. (2016). Prevalence of Temporomandibular Disorder Pain in Chinese Adolescents Compared to an Age-Matched Swedish Population. J. Oral Rehabil..

[76] Akhter R., Hassan N.M.M., Nameki H., Nakamura K., Honda O., Morita M. (2004). Association of Dietary Habits with Symptoms of Temporomandibular Disorders in Bangladeshi Adolescents. J. Oral Rehabil..

[77] Nguyen M.S., Reemann P., Loorits D., Lives P., Jagomägi T., Nguyen T., Saag M., Voog-Oras U. (2019). Association of Temporomandibular Joint Osseous Changes with Anxiety, Depression, and Limitation of Mandibular Function in Elderly Vietnamese. East Asian Arch. Psychiatry.

[78] Nguyen M.S., Jagomägi T., Nguyen T., Saag M., Voog-Oras Ü. (2017). Symptoms and Signs of Temporomandibular Disorders among Elderly Vietnamese. Proc. Singap. Healthc..

[79] Chauhan D., Kaundal J., Karol S., Chauhan T. (2013). Prevalence of Signs and Symptoms of Temporomandibular Disorders in Urban and Rural Children of Northern Hilly State, Himachal Pradesh, India: A Cross Sectional Survey. Dent. Hypotheses.

[80] Balke Z., Rammelsberg P., Leckel M., Schmitter M. (2010). Prevalence of Temporomandibular Disorders: Samples Taken from Attendees of Medical Health-Care Centers in the Islamic Republic of Iran. J. Orofac. Pain.

[81] Stone P.W. (2002). Popping the (PICO) Question in Research and Evidence-Based Practice. Appl. Nurs. Res..

[82] Frandsen T.F., Bruun Nielsen M.F., Lindhardt C.L., Eriksen M.B. (2020). Using the Full PICO Model as a Search Tool for Systematic Reviews Resulted in Lower Recall for Some PICO Elements. J. Clin. Epidemiol..

[83] Rzhetsky A., Nei M. (1992). Statistical Properties of the Ordinary Least-Squares, Generalized Least-Squares, and Minimum-Evolution Methods of Phylogenetic Inference. J. Mol. Evol..

[84] Kilmer J.T., Rodríguez R.L. (2017). Ordinary Least Squares Regression Is Indicated for Studies of Allometry. J. Evol. Biol..

[85] Ding S. (2021). Identifying Influential Data Points With Cook’s Distance. Medium.

[86] Muller K.E., Mok M.C. (1997). The Distribution of Cook’s D Statistic. Commun. Stat. Theory Methods.

[87] Nations U. 68% of the World Population Projected to Live in Urban Areas by 2050, Says UN. https://www.un.org/uk/desa/68-world-population-projected-live-urban-areas-2050-says-un.

[88] Prevalence of TMJD and Its Signs and Symptoms|Data & Statistics|National Institute of Dental and Craniofacial Research. https://www.nidcr.nih.gov/research/data-statistics/facial-pain/prevalence.

[89] Schuster S., Stark H. (2014). What Can We Learn from Einstein and Arrhenius about the Optimal Flow of Our Blood?. Biochim. Biophys. Acta.

[90] Bacaër N. (2011). Verhulst and the Logistic Equation (1838). A Short History of Mathematical Population Dynamics.

[91] Balak N., Inan D., Ganau M., Zoia C., Sönmez S., Kurt B., Akgül A., Tez M. (2021). A Simple Mathematical Tool to Forecast COVID-19 Cumulative Case Numbers. Clin. Epidemiol. Glob. Health.

[92] Naeem M., Ozuem W., Howell K., Ranfagni S. (2024). Demystification and Actualisation of Data Saturation in Qualitative Research Through Thematic Analysis. Int. J. Qual. Methods.

[93] Saunders B., Sim J., Kingstone T., Baker S., Waterfield J., Bartlam B., Burroughs H., Jinks C. (2018). Saturation in Qualitative Research: Exploring Its Conceptualization and Operationalization. Qual. Quant..

[94] Guest G., Namey E., Chen M. (2020). A Simple Method to Assess and Report Thematic Saturation in Qualitative Research. PLoS ONE.

[95] Minervini G., Franco R., Marrapodi M.M., Fiorillo L., Cervino G., Cicciù M. (2023). Prevalence of Temporomandibular Disorders in Children and Adolescents Evaluated with Diagnostic Criteria for Temporomandibular Disorders: A Systematic Review with Meta-Analysis. J. Oral Rehabil..

[96] Lei J., Fu J., Yap A.U.J., Fu K.-Y. (2016). Temporomandibular Disorders Symptoms in Asian Adolescents and Their Association with Sleep Quality and Psychological Distress. CRANIO®.

[97] De Oliveira A.S., Dias E.M., Contato R.G., Berzin F. (2006). Prevalence Study of Signs and Symptoms of Temporomandibular Disorder in Brazilian College Students. Braz. Oral Res..

[98] Projections by Continent-World Projections-Data. https://www.ined.fr/en/everything_about_population/data/world-projections/projections-by-continent/.

[99] Viechtbauer W. (2010). Conducting Meta-Analyses in R with the Metafor Package. J. Stat. Softw..

[100] Wickham H., François R., Henry L., Müller K., Vaughan D. (2023). dplyr: A Grammar of Data Manipulation. https://www.scirp.org/reference/referencespapers?referenceid=3610085.

[101] Wickham H. (2016). Ggplot2: Elegant Graphics for Data Analysis.

[102] readxl: Read Excel Files 2023. https://CRAN.R-project.org/package=readxl.

[103] Wickham H., Seidel D. (2022). RStudio Scales: Scale Functions for Visualization. https://CRAN.R-project.org/package=scales.

[104] Wickham H., Vaughan D., Girlich M., Ushey K., Posit Software, PBC (2024). Tidyr: Tidy Messy Data. https://cran.r-project.org/web/packages/tidyr/index.html.

[105] Wickham H., Henry L., Posit Software, PBC (2025). [cph; fnd Purrr: Functional Programming Tools. https://cran.r-project.org/web/packages/purrr/index.html.

[106] Wieckiewicz M., Grychowska N., Nahajowski M., Hnitecka S., Kempiak K., Charemska K., Balicz A., Chirkowska A., Zietek M., Winocur E. (2020). Prevalence and Overlaps of Headaches and Pain-Related Temporomandibular Disorders Among the Polish Urban Population. J. Oral Facial Pain Headache.

[107] Vompi C., Serritella E., Galluccio G., Pistella S., Segnalini A., Giannelli L., Di Paolo C. (2020). Evaluation of Vision in Gnathological and Orthodontic Patients with Temporomandibular Disorders: A Prospective Experimental Observational Cohort Study. J. Int. Soc. Prev. Community Dent..

[108] Zieliński G., Filipiak Z., Ginszt M., Matysik-Woźniak A., Rejdak R., Gawda P. (2021). The Organ of Vision and the Stomatognathic System—Review of Association Studies and Evidence-Based Discussion. Brain Sci..

[109] Marchesi A., Bellini D., Pellegrini C., Rizzi A., Marchesi R., Sardella A. (2024). The Influence of Temporomandibular Joint Disorders and Mandibular Position on Visual Capacities: A Case-Control Study. J. Int. Soc. Prev. Community Dent..

[110] Marchili N., Ortu E., Pietropaoli D., Cattaneo R., Monaco A. (2016). Dental Occlusion and Ophthalmology: A Literature Review. Open Dent. J..

[111] Zieliński G., Matysik-Woźniak A., Baszczowski M., Rapa M., Ginszt M., Pająk B., Szkutnik J., Rejdak R., Gawda P. (2023). Myopia & Painful Muscle Form of Temporomandibular Disorders: Connections between Vision, Masticatory and Cervical Muscles Activity and Sensitivity and Sleep Quality. Sci. Rep..

[112] Getzell J.H., and Cutler M.J. (2023). Vision—The Missing Link. CRANIO®.

[113] Natu V.P., Yap A.U.-J., Su M.H., Irfan Ali N.M., Ansari A. (2018). Temporomandibular Disorder Symptoms and Their Association with Quality of Life, Emotional States and Sleep Quality in South-East Asian Youths. J. Oral Rehabil..

[114] Figueiredo Ribeiro D.C., Ferreira Gradella C.M., Franco Rocha Rodrigues L.L., Abanto J., Oliveira L.B. (2020). The Impact of Temporomandibular Disorders on the Oral Health-Related Quality of Life of Brazilian Children: A Cross-Sectional Study. J. Dent. Child. Chic. Ill.

[115] Camacho J.G.D.D., Oltramari-Navarro P.V.P., Navarro R.d.L., Conti A.C.d.C.F., Conti M.R.d.A., Marchiori L.L.d.M., Fernandes K.B.P. (2014). Signs and Symptoms of Temporomandibular Disorders in the Elderly. CoDAS.

[116] Mendiburu-Zavala C.E., Castillero-Rosas A.S., Lugo-Ancona P.E., Carrillo-Mendiburu J. (2020). Temporomandibular Dysfunction and Depression in Adolescents of Mayan Ancestry. Bol. Med. Hosp. Infant. Mex..

[117] Alshahrani A.A., Saini R.S., Okshah A., Alshadidi A.A.F., Kanji M.A., Vyas R., Binduhayyim R.I.H., Ahmed N., Mosaddad S.A., Heboyan A. (2024). The Association between Genetic Factors and Temporomandibular Disorders: A Systematic Literature Review. Arch. Oral Biol..

[118] Smith S.B., Mir E., Bair E., Slade G.D., Dubner R., Fillingim R.B., Greenspan J.D., Ohrbach R., Knott C., Weir B. (2013). Genetic Variants Associated with Development of TMD and Its Intermediate Phenotypes: The Genetic Architecture of TMD in the OPPERA Prospective Cohort Study. J. Pain.

[119] Melis M., Di Giosia M. (2016). The Role of Genetic Factors in the Etiology of Temporomandibular Disorders: A Review. CRANIO®.

[120] de Araujo R.P., Groppo F.C., Ferreira L.E.N., Guimarães A.S., Figueroba S.R. (2015). Correlation between Facial Types and Muscle TMD in Women: An Anthropometric Approach. Braz. Oral Res..

[121] World Bank and WHO: Half the World Lacks Access to Essential Health Services, 100 Million Still Pushed into Extreme Poverty Because of Health Expenses. https://www.who.int/news/item/13-12-2017-world-bank-and-who-half-the-world-lacks-access-to-essential-health-services-100-million-still-pushed-into-extreme-poverty-because-of-health-expenses.

[122] National Academies of Sciences, Engineering, and Medicine, Health and Medicine Division, Board on Health Care Services, Board on Global Health, Committee on Improving the Quality of Health Care Globally (2018). The Current State of Global Health Care Quality. Crossing the Global Quality Chasm: Improving Health Care Worldwide.

[123] Morales M.E., Yong R.J. (2021). Racial and Ethnic Disparities in the Treatment of Chronic Pain. Pain Med. Malden Mass.

[124] Meints S.M., Cortes A., Morais C.A., Edwards R.R. (2019). Racial and Ethnic Differences in the Experience and Treatment of Noncancer Pain. Pain Manag..

[125] Zimmer Z., Fraser K., Grol-Prokopczyk H., Zajacova A. (2022). A Global Study of Pain Prevalence across 52 Countries: Examining the Role of Country-Level Contextual Factors. Pain.

[126] Kim H.J., Yang G.S., Greenspan J.D., Downton K.D., Griffith K.A., Renn C.L., Johantgen M., Dorsey S.G. (2017). Racial and Ethnic Differences in Experimental Pain Sensitivity: Systematic Review and Meta-Analysis. Pain.

[127] Zhang Y., Baik S.H., Fendrick A.M., Baicker K. (2012). Comparing Local and Regional Variation in Health Care Spending. N. Engl. J. Med..

[128] Enyeji A.M., Barengo N.C., Ramirez G., Ibrahimou B., Arrieta A. (2023). Regional Variation in Health Care Utilization Among Adults With Inadequate Cardiovascular Health in the USA. Cureus.

[129] Meisters R., Westra D., Putrik P., Bosma H., Ruwaard D., Jansen M. (2022). Regional Differences in Healthcare Costs Further Explained: The Contribution of Health, Lifestyle, Loneliness and Mastery. TSG Tijdschr. Voor Gezondheidswetenschappen.

[130] Garstka A.A., Kozowska L., Kijak K., Brzózka M., Gronwald H., Skomro P., Lietz-Kijak D. (2023). Accurate Diagnosis and Treatment of Painful Temporomandibular Disorders: A Literature Review Supplemented by Own Clinical Experience. Pain Res. Manag..

[131] Zieliński G., Gawda P. (2024). Surface Electromyography in Dentistry—Past, Present and Future. J. Clin. Med..

[132] Liu F., Steinkeler A. (2013). Epidemiology, Diagnosis, and Treatment of Temporomandibular Disorders. Dent. Clin. N. Am..

[133] Shrivastava M., Ye L. (2023). Neuroimaging and Artificial Intelligence for Assessment of Chronic Painful Temporomandibular Disorders—A Comprehensive Review. Int. J. Oral Sci..

[134] Mehta V., Tripathy S., Noor T., Mathur A. (2025). Artificial Intelligence in Temporomandibular Joint Disorders: An Umbrella Review. Clin. Exp. Dent. Res..

[135] Jha N., Lee K., Kim Y.-J. (2022). Diagnosis of Temporomandibular Disorders Using Artificial Intelligence Technologies: A Systematic Review and Meta-Analysis. PLoS ONE.

[136] Almășan O., Leucuța D.-C., Hedeșiu M., Mureșanu S., Popa Ș.L. (2023). Temporomandibular Joint Osteoarthritis Diagnosis Employing Artificial Intelligence: Systematic Review and Meta-Analysis. J. Clin. Med..

[137] Manek M., Maita I., Bezerra Silva D.F., Pita de Melo D., Major P.W., Jaremko J.L., Almeida F.T. (2025). Temporomandibular Joint Assessment in MRI Images Using Artificial Intelligence Tools: Where Are We Now? A Systematic Review. Dento Maxillo Facial Radiol..

