# Supplementary material for: Quo Vadis Temporomandibular Disorders? By 2050, the Global Prevalence of TMD May Approach 44%
Source: J Clin Med. 2025 Jun 20;14(13):4414. doi: 10.3390/jcm14134414 (PMC12249499; doi:10.3390/jcm14134414)
Supplement: Supplementary file 1 [file jcm-14-04414-s001.zip › Supplementary Material S1 – Exported Data.pdf]

# Quo Vadis Temporomandibular Disorders? By 2050, the Global Prevalence of TMD May Approach 44%

**Grzegorz Zieliński**

Department of Sports Medicine, Medical University of Lublin, 20-093 Lublin, Poland; grzegorz.zielinski@umlub.pl

Data exported from study [1].
Detailed results are presented for the following regions:

Global

| *Age* | *Proportion* |
| --- | --- |
| Up to 18 yrs. | 0.27 |
| 18-60 yrs. | 0.41 |
| 60+ yrs. | 0.36 |

Asia

| *Age* | *Proportion* |
| --- | --- |
| Up to 18 yrs. | 0.27 |
| 18-60 yrs. | 0.41 |
| 60+ yrs. | 0.36 |

North America

| *Age* | *Proportion* |
| --- | --- |
| Up to 18 yrs. | 0.37 |
| 18-60 yrs. | - |
| 60+ yrs. | - |

South America

| *Age* | *Proportion* |
|---|---|
| Up to 18 yrs. | 0.33 |
| 18-60 yrs. | 0.56 |
| 60+ yrs. | 0.56 |

Europe

| *Age* | *Proportion* |
|---|---|
| Up to 18 yrs. | 0.18 |
| 18-60 yrs. | 0.41 |
| 60+ yrs. | 0.32 |

**References**


1. Zieliński, G.; Pająk-Zielińska, B.; Ginszt, M. A Meta-Analysis of the Global Prevalence of Temporomandibular Disorders. *J. Clin. Med.* **2024**, *13*, 1365, doi:10.3390/jcm13051365.
