# Supplementary material for: Quo Vadis Temporomandibular Disorders? By 2050, the Global Prevalence of TMD May Approach 44%
Source: J Clin Med. 2025 Jun 20;14(13):4414. doi: 10.3390/jcm14134414 (PMC12249499; doi:10.3390/jcm14134414)
Supplement: Supplementary file 1 [file jcm-14-04414-s001.zip › Supplementary Material S2 - Analysis of the Impact of Urbanisation on TMD Prevalence.pdf]

Quo Vadis Temporomandibular Disorders? By 2050, the Global Prevalence of TMD May Approach 44%

Grzegorz Zieliński

Department of Sports Medicine, Medical University of Lublin, 20-093 Lublin, Poland; grzegorz.zielinski@umlub.pl

Due to a lack of clear studies examining the effect of urbanisation on temporomandibular disorders (TMD) prevalence, a systematic search of the PubMed and Scopus databases was conducted [1–3]. The keywords used were: "TMD", "temporomandibular disorder", and "rural", with no date restrictions. Of 190 identified articles, 66 abstracts were reviewed, and 18 full texts were analysed. Due to the inability to export data based on urban or rural regions, the following studies were excluded after full-text assessment: [4–8]. Ultimately, 13 studies [9–21] comparing TMD prevalence in urban and rural populations were included (Figure S1).
The summary of the PICO standards (population, intervention, comparison, outcome) [22,23], is found in Table S1.

Table S1. PICO Summary.

P (Population)	I (Intervention / Exposure)	C (Comparison)	O (Outcome)
Individuals from urban and rural populations studied for TMD prevalence.	Urbanisation (percentage of population living in urban areas).	Populations with different levels of urbanisation (rural vs urban regions).	Prevalence of temporomandibular disorders.

Urbanisation rates (%) corresponding to the year of data collection were assigned based on literature values [24]. Studies were categorised by continent: America, Europe, and Asia. For each, linear regression (OLS – ordinary least squares) [25,26] was used to model the relationship between urbanisation (%) and TMD prevalence (%). Influential observations were removed using Cook's distance (>4/n) [27,28]. Coefficients of determination (R^2) were calculated, and regression coefficients were interpreted as the change in TMD prevalence per 1% increase in urbanisation.

Analyses were conducted using the R Statistical language (version 4.1.1; R Core Team, 2021) on Windows 10 Pro 64 bit (build 19045), using the packages *metafor* (version 3.8.1; [29]), *dplyr* (version 1.1.2; [30]), *ggplot2* (version 3.4.0; [31]), readxl (version 1.4.5; [32]), scales (version 1.4.0; [33]), tidyr (version 1.3.1; [34]), purr (version 1.0.4; [35]).

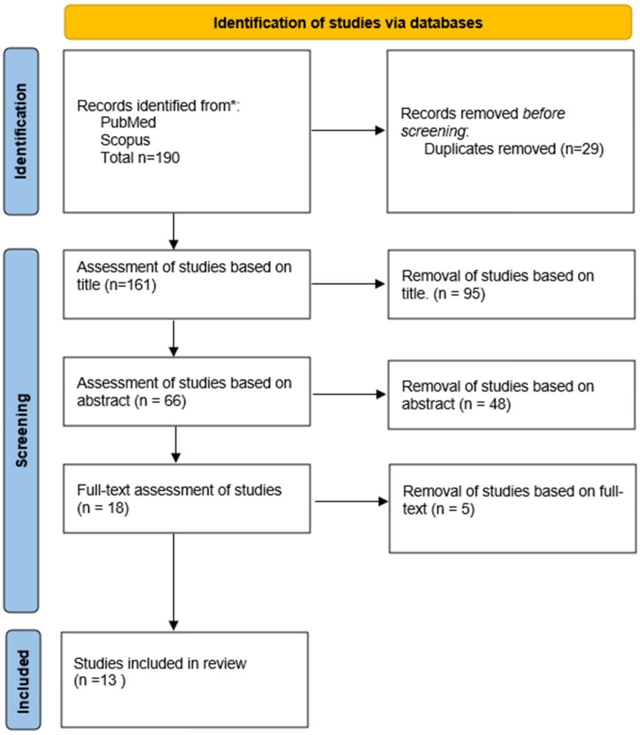

Figure S1. PRISMA flow diagram.

Results

The following studies were included in the analysis: [9–21] (Table S2).

Table S2. Detailed presentation of the results.

Source	Area	Estimated Urbanization Rate (%)	TMD Prevalence (%)	Continent
Restrepo et al. [9] 2021	Urban	82	20	Americas
	Rural	18	31	Americas
Costa et al. [10] 2018	Urban	87	33	Americas
	Rural	13	33	Americas
Goddard and Karibe [11] 2001	Urban	79	21	Americas
	Rural	21	14	Americas
Nilsson et al.[12] 2000	Urban	84	4	Europe
	Rural	16	4	Europe
Østensjø et al. 2013	Urban	80	9	Europe
	Rural	20	3	Europe
Kim et al. [14] 2019	Urban	81	25	Asia
	Rural	19	26	Asia
Shenoy et al. [15] 2020	Urban	35	1	Asia
	Rural	65	4	Asia
Hongxing et al. [16]	Urban	36	17	Asia
	Rural	64	12	Asia

2000				
Akhter et al. [17] 2004	Urban	26	21	Asia
	Rural	74	30	Asia
Nguyen et al. [18] 2017	Urban	35	49	Asia
	Rural	65	51	Asia
Nguyen et al. [19] 2017	Urban	35	41	Asia
	Rural	65	60	Asia
Chauhan et al. [20] 2013	Urban	32	3	Asia
	Rural	68	2	Asia
Balke et al. [21] 2010	Urban	71	20	Asia
	Rural	29	46	Asia

The analysis of the relationship between the level of urbanisation and the prevalence of TMD was conducted separately for three continents: America, Europe, and Asia. Due to data limitations, it was not possible to distinguish between North and South America, and no data were found for Africa or Australia. Therefore, for the latter two continents, a global indicator was constructed.

In the case of America, the regression coefficient was −0.023, indicating a decrease in TMD prevalence by 0.023 percentage points for every 1% increase in urbanisation. However, the coefficient of determination (R^2) was only 0.011, suggesting a very low explanatory power of the model. No influential observations were identified. Consequently, the model suggests only a slight negative correlation between urbanisation and TMD prevalence, but this relationship is statistically weak and not reliable.

In Europe, the regression coefficient was close to zero (~0.000), indicating virtually no effect of urbanisation on TMD. The R^2 value was negative infinity (−∞), an artefactual result caused by a lack of variability in the data – both urban and rural areas reported similar TMD values. No influential observations were found. Due to the homogeneity of the data, the model proved non-functional, and the available information does not support a significant association between urbanisation and TMD in Europe.

In contrast, a more pronounced relationship was observed in Asia. The regression coefficient was −0.316, meaning that each 1% increase in urbanisation was associated with a 0.316 percentage point decrease in TMD prevalence. The R^2 value reached 0.395, indicating a moderate explanatory power of the model. One influential observation was identified – Akhter et al. [17], with urbanisation at 26% and TMD prevalence at 21% – and was excluded from the analysis. These results suggest a significant negative correlation between urbanisation and the risk of TMD in Asian populations: the higher the level of urbanisation, the lower the likelihood of developing TMD.

Subsequently, a global indicator was calculated. The results of the regression analysis showed a coefficient of −0.0034, indicating that each 1% increase in urbanisation was associated with a 0.0034 percentage point decrease in TMD prevalence. This is a very small value, likely lacking clinical significance. The coefficient of determination (R^2) was 0.000027, meaning that urbanisation explained only 0.003% of the variability in TMD prevalence in the analysed sample. Such a low value indicates a lack of statistically significant association between these variables.

Conclusions

Urbanisation may exert varying effects on the prevalence of TMD depending on the geographical region. The strongest association has been observed in Asian countries, where an increase in urbanisation levels correlated with a statistically significant decrease in TMD prevalence. In the Americas and Europe, this relationship was weak or not demonstrated. On a global scale, the analysis did not confirm a significant impact of urbanisation on TMD epidemiology, which may suggest the importance of environmental and cultural determinants in its aetiopathogenesis.

References

1. Zieliński, G.; Pająk, A.; Wójcicki, M. Global Prevalence of Sleep Bruxism and Awake Bruxism in Pediatric and Adult Populations: A Systematic Review and Meta-Analysis. *J. Clin. Med.* **2024**, *13*, 4259, doi:10.3390/jcm13144259.
2. Zieliński, G.; Pająk-Zielińska, B. Association between Estrogen Levels and Temporomandibular Disorders: An Updated Systematic Review. *Int. J. Mol. Sci.* **2024**, *25*, 9867, doi:10.3390/ijms25189867.
3. Berger, M.; Szalewski, L.; Bakalczuk, M.; Bakalczuk, G.; Bakalczuk, S.; Szkutnik, J. Association between Estrogen Levels and Temporomandibular Disorders: A Systematic Literature Review. *Menopause Rev. Menopauzalny* **2015**, *14*, 260–270, doi:10.5114/pm.2015.56538.
4. Michalak, M.; Paulo, M.; Bożyk, A.; Zadrożny, Ł.; Wysokińska-Miszczuk, J.; Michalak, I.; Borowicz, J. Incidence of Abnormalities in Temporomandibular Joints in a Population of 1,100 Urban and Rural Patients Lacking Teeth and Other Parafunctions in 2003-2008. An International Problem. *Ann. Agric. Environ. Med. AAEM* **2013**, *20*, 86–90.
5. Montero, J.; Llodra, J.-C.; Bravo, M. Prevalence of the Signs and Symptoms of Temporomandibular Disorders Among Spanish Adults and Seniors According to Five National Surveys Performed Between 1993 and 2015. *J. Oral Facial Pain Headache* **2018**, *32*, 349–357, doi:10.11607/ofph.2085.
6. Mejersjö, C.; Ovesson, D.; Mossberg, B. Oral Parafunctions, Piercing and Signs and Symptoms of Temporomandibular Disorders in High School Students. *Acta Odontol. Scand.* **2016**, *74*, 279–284, doi:10.3109/00016357.2015.1114668.
7. Simangwa, L.D.; Johansson, A.-K.; Johansson, A.; Minja, I.K.; Åstrøm, A.N. Oral Impacts on Daily Performances and Its Socio-Demographic and Clinical Distribution: A Cross-Sectional Study of Adolescents Living in Maasai Population Areas, Tanzania. *Health Qual. Life Outcomes* **2020**, *18*, 181, doi:10.1186/s12955-020-01444-7.
8. Fabian, F.M.; and Mumghamba, E.G.S. Risk Factors for Signs and Symptoms of TMD in a Rural Adult Southeast Tanzanian Population. *CRANIO®* **2008**, *26*, 44–49, doi:10.1179/crn.2008.006.
9. Restrepo, C.; Ortiz, A.M.; Henao, A.C.; Manrique, R. Association between Psychological Factors and Temporomandibular Disorders in Adolescents of Rural and Urban Zones. *BMC Oral Health* **2021**, *21*, 140, doi:10.1186/s12903-021-01485-4.
10. Costa, M.J.F.; Lins, C.A. de A.; Macedo, L.P.V. de; Sousa, V.P.S. de; Duque, J.A.; Souza, M.C. de Clinical and Self-Perceived Oral Health Assessment of Elderly Residents in Urban, Rural, and Institutionalized Communities. *Clin. Sao Paulo Braz.* **2019**, *74*, e972, doi:10.6061/clinics/2019/e972.
11. Goddard, G.; and Karibe, H. TMD Prevalence in Rural and Urban Native American Populations. *CRANIO®* **2002**, *20*, 125–128, doi:10.1080/08869634.2002.11746201.
12. Nilsson, I.-M.; List, T.; Drangsholt, M. Prevalence of Temporomandibular Pain and Subsequent Dental Treatment in Swedish Adolescents. *J. Orofac. Pain* **2005**, *19*, 144–150.
13. Østensjø, V.; Moen, K.; Storesund, T.; Rosén, A. Prevalence of Painful Temporomandibular Disorders and Correlation to Lifestyle Factors among Adolescents in Norway. *Pain Res. Manag.* **2017**, *2017*, 2164825, doi:10.1155/2017/2164825.
14. Kim, D.; Ko, S.-G.; Lee, E.-K.; Jung, B. The Relationship between Spinal Pain and Temporomandibular Joint Disorders in Korea: A Nationwide Propensity Score-Matched Study. *BMC Musculoskelet. Disord.* **2019**, *20*, 631, doi:10.1186/s12891-019-3003-4.
15. Shenoy, R.P.; Agrawal, R.; Abdul Salam, T.A.; Prashanth Shenoy, K. Screening for Temporomandibular Disorders and Other Oral Conditions among Adolescents in Mangaluru Taluk. *World J. Dent.* **2020**, *11*, 201–205, doi:10.5005/jp-journals-10015-1728.
16. Hongxing, L.; Astrøm, A.N.; List, T.; Nilsson, I.-M.; Johansson, A. Prevalence of Temporomandibular Disorder Pain in Chinese Adolescents Compared to an Age-Matched Swedish Population. *J. Oral Rehabil.* **2016**, *43*, 241–248, doi:10.1111/joor.12366.
17. Akhter, R.; Hassan, N.M.M.; Nameki, H.; Nakamura, K.; Honda, O.; Morita, M. Association of Dietary Habits with Symptoms of Temporomandibular Disorders in Bangladeshi Adolescents. *J. Oral Rehabil.* **2004**, *31*, 746–753, doi:10.1111/j.1365-2842.2004.01420.x.

18. Nguyen, M.S.; Reemann, P.; Loorits, D.; Lives, P.; Jagomägi, T.; Nguyen, T.; Saag, M.; Voog-Oras, U. Association of Temporomandibular Joint Osseous Changes with Anxiety, Depression, and Limitation of Mandibular Function in Elderly Vietnamese. *East Asian Arch. Psychiatry* **2019**, *29*, 20–25, doi:10.12809/eaap1749.
19. Nguyen, M.S.; Jagomägi, T.; Nguyen, T.; Saag, M.; Voog-Oras, Ü. Symptoms and Signs of Temporomandibular Disorders among Elderly Vietnamese. *Proc. Singap. Healthc.* **2017**, *26*, 211–216, doi:10.1177/2010105817694907.
20. Chauhan, D.; Kaundal, J.; Karol, S.; Chauhan, T. Prevalence of Signs and Symptoms of Temporomandibular Disorders in Urban and Rural Children of Northern Hilly State, Himachal Pradesh, India: A Cross Sectional Survey. *Dent. Hypotheses* **2013**, *4*, 21, doi:10.4103/2155-8213.110182.
21. Balke, Z.; Rammelsberg, P.; Leckel, M.; Schmitter, M. Prevalence of Temporomandibular Disorders: Samples Taken from Attendees of Medical Health-Care Centers in the Islamic Republic of Iran. *J. Orofac. Pain* **2010**, *24*, 361–366.
22. Stone, P.W. Popping the (PICO) Question in Research and Evidence-Based Practice. *Appl. Nurs. Res.* **2002**, *15*, 197–198, doi:10.1053/apnr.2002.34181.
23. Frandsen, T.F.; Bruun Nielsen, M.F.; Lindhardt, C.L.; Eriksen, M.B. Using the Full PICO Model as a Search Tool for Systematic Reviews Resulted in Lower Recall for Some PICO Elements. *J. Clin. Epidemiol.* **2020**, *127*, 69–75, doi:10.1016/j.jclinepi.2020.07.005.
24. Nations, U. *Human Development Index*; United Nations;
25. Rzhetsky, A.; Nei, M. Statistical Properties of the Ordinary Least-Squares, Generalized Least-Squares, and Minimum-Evolution Methods of Phylogenetic Inference. *J. Mol. Evol.* **1992**, *35*, 367–375, doi:10.1007/BF00161174.
26. Kilmer, J.T.; Rodríguez, R.L. Ordinary Least Squares Regression Is Indicated for Studies of Allometry. *J. Evol. Biol.* **2017**, *30*, 4–12, doi:10.1111/jeb.12986.
27. Ding, S. Identifying Influential Data Points With Cook`s Distance. *Medium* 2021.
28. Muller, K.E.; Mok, M.C. THE DISTRIBUTION OF COOK'S D STATISTIC. *Commun. Stat. Theory Methods* **1997**, *26*, 10.1080/03610927708831932, doi:10.1080/03610927708831932.
29. Viechtbauer, W. Conducting Meta-Analyses in R with the Metafor Package. *J. Stat. Softw.* **2010**, *36*, doi:10.18637/jss.v036.i03.
30. Wickham, H.; François, R.; Henry, L.; Müller, K.; Vaughan, D.; Software, P.; PBC Dplyr: A Grammar of Data Manipulation 2023.
31. Wickham, H. *Ggplot2: Elegant Graphics for Data Analysis*; Use R!; 2nd ed. 2016.; Springer International Publishing : Imprint: Springer: Cham, 2016; ISBN 978-3-319-24277-4.
32. Wickham, H.; Bryan, J.; Posit; attribution), P. (Copyright holder of all R. code and all C. code without explicit copyright; code), M.K. (Author of included R.; code), K.V. (Author of included libxls; code), C.L. (Author of included libxls; code), B.C. (Author of included libxls; code), D.H. (Author of included libxls; code), E.M. (Author of included libxls Readxl: Read Excel Files 2023.
33. Wickham, H.; Seidel, D.; RStudio Scales: Scale Functions for Visualization 2022.
34. Wickham, H.; Vaughan, D.; Girlich, M.; Ushey, K.; Software, P.; PBC Tidyr: Tidy Messy Data 2024.
35. Wickham, H.; Henry, L.; Software, P.; PBC [cph; fnd Purrr: Functional Programming Tools 2025.
