# Supplementary material for: Quo Vadis Temporomandibular Disorders? By 2050, the Global Prevalence of TMD May Approach 44%
Source: J Clin Med. 2025 Jun 20;14(13):4414. doi: 10.3390/jcm14134414 (PMC12249499; doi:10.3390/jcm14134414)
Supplement: Supplementary file 1 [file jcm-14-04414-s001.zip › Supplementary Material S3 – Annual Growth Model.pdf]

Quo Vadis Temporomandibular Disorders? By 2050, the Global Prevalence of TMD May Approach 44%

Grzegorz Zieliński

Department of Sports Medicine, Medical University of Lublin, 20-093 Lublin, Poland; grzegorz.zielinski@umlub.pl

To model a realistic rate of TMD prevalence increase, an exponential fit function was developed, based on the approach by Holden et al. [1]. The model accounts for a decreasing growth rate as prevalence approaches upper biological limits near 100%. The function was calibrated so that, for a baseline prevalence of 12% [2,3], it yielded an annual growth rate of exactly 3.5% [2] Figure S1.

Final formula:

$$\Delta p_{annual} = 6.99 * e^{-0.057 * p_{2020}}$$

Where:

- Δp_{annual} = annual change in TMD prevalence (percentage points),
- p2020 = baseline TMD prevalence in the year 2020,
- e = Euler's number (approx. 2.718) [4].

Analyses were conducted using the R Statistical language (version 4.1.1; R Core Team, 2021) on Windows 10 Pro 64 bit (build 19045), using the packages *metafor* (version 3.8.1; [5]), *dplyr* (version 1.1.2; [6]), *ggplot2* (version 3.4.0; [7]), readxl (version 1.4.5; [8]), scales (version 1.4.0; [9]), tidyr (version 1.3.1; [10]), purr (version 1.0.4; [11]).

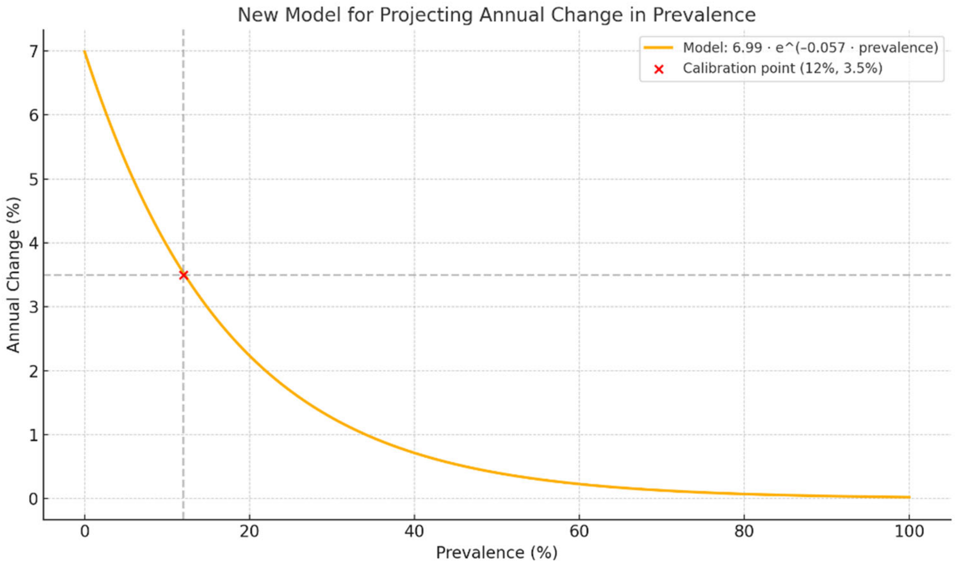

Figure S1. Calibration Point.

References

1. Holden, B.A.; Fricke, T.R.; Wilson, D.A.; Jong, M.; Naidoo, K.S.; Sankaridurg, P.; Wong, T.Y.; Naduvilath, T.J.; Resnikoff, S. Global Prevalence of Myopia and High Myopia and Temporal Trends from 2000 through 2050. *Ophthalmology* **2016**, *123*, 1036–1042, doi:10.1016/j.ophtha.2016.01.006.
2. Bair, E.; Brownstein, N.C.; Ohrbach, R.; Greenspan, J.D.; Dubner, R.; Fillingim, R.B.; Maixner, W.; Smith, S.; Diatchenko, L.; Gonzalez, Y.; et al. Study Protocol, Sample Characteristics, and Loss to Follow-Up: The OPPERA Prospective Cohort Study. *J. Pain Off. J. Am. Pain Soc.* **2013**, *14*, 10.1016/j.jpain.2013.06.006, doi:10.1016/j.jpain.2013.06.006.
3. Prevalence of TMJD and Its Signs and Symptoms | Data & Statistics | National Institute of Dental and Craniofacial Research Available online: https://www.nidcr.nih.gov/research/data-statistics/facial-pain/prevalence (accessed on 8 May 2025).
4. Schuster, S.; Stark, H. What Can We Learn from Einstein and Arrhenius about the Optimal Flow of Our Blood? *Biochim. Biophys. Acta* **2014**, *1840*, 271–276, doi:10.1016/j.bbagen.2013.08.026.
5. Viechtbauer, W. Conducting Meta-Analyses in R with the Metafor Package. *J. Stat. Softw.* **2010**, *36*, doi:10.18637/jss.v036.i03.
6. Wickham, H.; François, R.; Henry, L.; Müller, K.; Vaughan, D.; Software, P.; PBC Dplyr: A Grammar of Data Manipulation 2023.
7. Wickham, H. *Ggplot2: Elegant Graphics for Data Analysis*; Use R!; 2nd ed. 2016.; Springer International Publishing : Imprint: Springer: Cham, 2016; ISBN 978-3-319-24277-4.
8. Wickham, H.; Bryan, J.; Posit; attribution), P. (Copyright holder of all R. code and all C. code without explicit copyright; code), M.K. (Author of included R.; code), K.V. (Author of included libxls; code), C.L. (Author of included libxls; code), B.C. (Author of included libxls; code), D.H. (Author of included libxls; code), E.M. (Author of included libxls Readxl: Read Excel Files 2023.
9. Wickham, H.; Seidel, D.; RStudio Scales: Scale Functions for Visualization 2022.
10. Wickham, H.; Vaughan, D.; Girlich, M.; Ushey, K.; Software, P.; PBC Tidyr: Tidy Messy Data 2024.
11. Wickham, H.; Henry, L.; Software, P.; PBC [cph; fnd Purrr: Functional Programming Tools 2025.
