# Supplementary material for: Quo Vadis Temporomandibular Disorders? By 2050, the Global Prevalence of TMD May Approach 44%
Source: J Clin Med. 2025 Jun 20;14(13):4414. doi: 10.3390/jcm14134414 (PMC12249499; doi:10.3390/jcm14134414)
Supplement: Supplementary file 1 [file jcm-14-04414-s001.zip › Supplementary Material S4 – Detailed Results.pdf]

Quo Vadis Temporomandibular Disorders? By 2050, the Global Prevalence of TMD May Approach 44%

Grzegorz Zieliński

Department of Sports Medicine, Medical University of Lublin, 20-093 Lublin, Poland; grzegorz.zielinski@umlub.pl

Region	Age Group	Year	Projected Proportion	95% CI Lower	95% CI Upper
Africa	0-18	2030	0.31	0.26	0.36
Africa	0-18	2040	0.35	0.30	0.40
Africa	0-18	2050	0.40	0.35	0.45
Africa	0-18	2075	0.45	0.40	0.50
Africa	0-18	2100	0.49	0.44	0.54
Africa	18-60	2030	0.43	0.38	0.48
Africa	18-60	2040	0.46	0.41	0.51
Africa	18-60	2050	0.49	0.44	0.54
Africa	18-60	2075	0.52	0.47	0.57
Africa	18-60	2100	0.55	0.50	0.60
Africa	60+	2030	0.37	0.32	0.42
Africa	60+	2040	0.39	0.34	0.44
Africa	60+	2050	0.42	0.37	0.47
Africa	60+	2075	0.45	0.40	0.50
Africa	60+	2100	0.48	0.43	0.53
Asia	0-18	2030	0.29	0.24	0.34
Asia	0-18	2040	0.32	0.27	0.37
Asia	0-18	2050	0.34	0.29	0.39
Asia	0-18	2075	0.37	0.32	0.42
Asia	0-18	2100	0.39	0.34	0.44
Asia	18-60	2030	0.42	0.37	0.47
Asia	18-60	2040	0.42	0.37	0.47
Asia	18-60	2050	0.42	0.37	0.47
Asia	18-60	2075	0.44	0.39	0.49
Asia	18-60	2100	0.45	0.40	0.50
Asia	60+	2030	0.36	0.31	0.41
Asia	60+	2040	0.36	0.31	0.41
Asia	60+	2050	0.35	0.30	0.40
Asia	60+	2075	0.36	0.31	0.41
Asia	60+	2100	0.37	0.32	0.42
Australia	0-18	2030	0.31	0.26	0.36
Australia	0-18	2040	0.35	0.30	0.40
Australia	0-18	2050	0.40	0.35	0.45
Australia	0-18	2075	0.45	0.40	0.50
Australia	0-18	2100	0.49	0.44	0.54
Australia	18-60	2030	0.43	0.38	0.48

Australia	18-60	2040	0.46	0.41	0.51
Australia	18-60	2050	0.49	0.44	0.54
Australia	18-60	2075	0.52	0.47	0.57
Australia	18-60	2100	0.55	0.50	0.60
Australia	60+	2030	0.37	0.32	0.42
Australia	60+	2040	0.39	0.34	0.44
Australia	60+	2050	0.42	0.37	0.47
Australia	60+	2075	0.45	0.40	0.50
Australia	60+	2100	0.48	0.43	0.53
Europe	0-18	2030	0.25	0.20	0.30
Europe	0-18	2040	0.32	0.27	0.37
Europe	0-18	2050	0.38	0.33	0.43
Europe	0-18	2075	0.44	0.39	0.49
Europe	0-18	2100	0.48	0.43	0.53
Europe	18-60	2030	0.43	0.38	0.48
Europe	18-60	2040	0.46	0.41	0.51
Europe	18-60	2050	0.49	0.44	0.54
Europe	18-60	2075	0.52	0.47	0.57
Europe	18-60	2100	0.55	0.50	0.60
Europe	60+	2030	0.34	0.29	0.39
Europe	60+	2040	0.37	0.32	0.42
Europe	60+	2050	0.40	0.35	0.45
Europe	60+	2075	0.44	0.39	0.49
Europe	60+	2100	0.47	0.42	0.52
North America	0-18	2030	0.38	0.33	0.43
North America	0-18	2040	0.41	0.36	0.46
North America	0-18	2050	0.43	0.38	0.48
North America	0-18	2075	0.46	0.41	0.51
North America	0-18	2100	0.50	0.45	0.55
North America	18-60	2030	0.43	0.38	0.48
North America	18-60	2040	0.46	0.41	0.51
North America	18-60	2050	0.48	0.43	0.53
North America	18-60	2075	0.52	0.47	0.57
North America	18-60	2100	0.54	0.49	0.59
North America	60+	2030	0.37	0.32	0.42
North America	60+	2040	0.39	0.34	0.44
North America	60+	2050	0.41	0.36	0.46
North America	60+	2075	0.44	0.39	0.49
North America	60+	2100	0.47	0.42	0.52
South America	0-18	2030	0.35	0.30	0.40
South America	0-18	2040	0.38	0.33	0.43
South America	0-18	2050	0.41	0.36	0.46
South America	0-18	2075	0.45	0.40	0.50
South America	0-18	2100	0.49	0.44	0.54
South America	18-60	2030	0.56	0.51	0.61
South America	18-60	2040	0.56	0.51	0.61

South America	18-60	2050	0.56	0.51	0.61
South America	18-60	2075	0.57	0.52	0.62
South America	18-60	2100	0.57	0.52	0.62
South America	60+	2030	0.56	0.51	0.61
South America	60+	2040	0.56	0.51	0.61
South America	60+	2050	0.56	0.51	0.61
South America	60+	2075	0.56	0.51	0.61
South America	60+	2100	0.56	0.51	0.61

Region	Year	Projected Proportion	95% CI Lower	95% CI Upper
Africa	2030	0.37	0.32	0.42
Africa	2040	0.40	0.35	0.45
Africa	2050	0.43	0.38	0.48
Africa	2075	0.47	0.42	0.52
Africa	2100	0.51	0.46	0.56
Asia	2030	0.36	0.31	0.41
Asia	2040	0.36	0.31	0.41
Asia	2050	0.37	0.32	0.42
Asia	2075	0.39	0.34	0.44
Asia	2100	0.40	0.35	0.45
Australia	2030	0.37	0.32	0.42
Australia	2040	0.40	0.35	0.45
Australia	2050	0.43	0.38	0.48
Australia	2075	0.47	0.42	0.52
Australia	2100	0.51	0.46	0.56
Europe	2030	0.34	0.29	0.39
Europe	2040	0.38	0.33	0.43
Europe	2050	0.42	0.37	0.47
Europe	2075	0.47	0.42	0.52
Europe	2100	0.50	0.45	0.55
North America	2030	0.39	0.34	0.44
North America	2040	0.42	0.37	0.47
North America	2050	0.44	0.39	0.49
North America	2075	0.47	0.42	0.52
North America	2100	0.50	0.45	0.55
South America	2030	0.49	0.44	0.54
South America	2040	0.50	0.45	0.55
South America	2050	0.51	0.46	0.56
South	2075	0.53	0.48	0.58

America				
South America	2100	0.54	0.49	0.59
Global	2030	0.39	0.34	0.44
Global	2040	0.41	0.36	0.46
Global	2050	0.44	0.39	0.49
Global	2075	0.47	0.42	0.52
Global	2100	0.49	0.44	0.54
